# Development and validation of quality of life scale of nasopharyngeal carcinoma patients: the QOL-NPC (version 2)

**DOI:** 10.1186/s12955-016-0480-0

**Published:** 2016-05-10

**Authors:** Yong Su, Chuan-wei Mo, Wan-qin Cheng, Lei Wang, Qian Xu, Zu-chun Wu, Zhe-li Wu, Li-zhi Liu, Xin-lin Chen

**Affiliations:** Department of Radiation Oncology, Cancer Centre, Sun Yat-sen University, Guangzhou, Guangdong Province China; Collaborative Innovation Center for Cancer Medicine, State Key Laboratory of Oncology in South China, Guangzhou, Guangdong Province China; Department of Biostatistics and Preventive Medicine, School of Basic Medical Sciences, Guangzhou University of Chinese Medicine, Guangzhou, Guangdong Province China; School of Basic Medical Sciences, Guangzhou University of Chinese Medicine, Guangzhou, Guangdong Province China

**Keywords:** Health-related quality of life, QOL-NPC, Reliability, Validity, Responsiveness

## Abstract

**Background:**

The aim was to develop and validate the quality of life scale for nasopharyngeal carcinoma (NPC) patients, the QOL-NPC (version 2), a specific instrument to measure quality of life for NPC patients.

**Methods:**

The QOL-NPC was developed and validated according to standard procedures. The patients were assessed using the QOL-NPC, FACT-G, and FACT-H&N. Classical test theory was used to evaluate the reliability, validity, and responsiveness of the QOL-NPC.

**Results:**

A total of 487 patients (97.4 %) completed the questionnaire. The QOL-NPC comprised four domains, as follows: physical function (eight items); psychological function (five items); social function (five items); and side effects (eight items). All of the items had a lower proportion of missing data. Cronbach's alpha values of the domains ranged from 0.72 to 0.84. The split-half reliability coefficients ranged from 0.77 to 0.84. All of the intra-class correlation coefficients were > 0.8. The normed fit index, non-normed fit index, and comparative fit index were >0.89. The root mean square error of approximation was 0.097, with a 90 % confidence interval (0.093, 0.100). The domain scores of the QOL-NPC were significantly correlated with the FACT-G and FACT-H&N (*P* < 0.05). All of the domain scores of patients using different amounts of radiotherapy were significantly different (*P* < 0.001). All domain scores decreased at the completion of radiotherapy, with effect sizes ranging from −0.82 to −0.22.

**Conclusions:**

The QOL-NPC is valid for measuring QOL with good reliability, validity, and responsiveness. The QOL-NPC is recommended to measure the QOL for Chinese NPC patients.

## Background

Nasopharyngeal carcinoma (NPC) is a malignancy with a high incidence in several geographic areas, especially southern China and Hong Kong [1, 2]. Because NPC is located in close proximity to the base of the skull and is sensitive to radiotherapy (RT), the primary treatment is RT alone or combined with chemotherapy [3, 4]. RT causes various side effects, such as xerostomia, dysphagia, and hearing loss. These side effects obviously have a serious impact on the health-related quality of life (QOL) in NPC patients. The QOL of the NPC patients has been widely studied with the following inventories: the European Organization for the Research and Treatment of Cancer Core QOL questionnaire (QLQ-C30) and head and neck module (QLQ-H&N35) [4, 5]; the MOS 36-item short-form health survey (SF-36) [6, 7]; The University of Washington Quality of Life [8]; the Functional Assessment of Cancer Therapy-General Scale (FACT-G) and head and neck module (FACT-H&N) [9, 10]; and the functional assessment of cancer therapy-nasopharyngeal (FACT-NP) [11].

The quality of life scale of nasopharyngeal carcinoma patients (QOL-NPC, version 1 [V1]) is an NPC-specific scale, which was used to assess the physical functioning and health status of the NPC people in the past 2 weeks [12–14]. The QOL-NPC was widely used to evaluate the QOL of Chinese NPC patients. Based on the application there were some problems. (1) The item of the QOL-NPC (V1) was rated on a 0–10 numeric visual analogue scale (VAS). Some patients reported that it was difficult to understand. For example, some patients with poorer reading skills were not able to distinguish score 5 from score 6. Most studies have reported that VAS and Likert responses have few differences in reliability and responsiveness, and are highly correlated [15–17]. Because the Likert responses are easier to administer, compute, and interpret for the patients, Likert responses are most often applied [15–18]. (2) Some items had problems. The previous patients reported that they were worried about the infection of the disease due to a lack of medical knowledge. Therefore, the item “worried about the infection of the disease” was applied in V1. Some important symptoms were missing in V1, such as pain in the throat and cough when swallowing food.

The purpose of this study was to develop and assess the QOL-NPC (version 2 [V2]) according to a set of standardized procedures of instrument development.

## Methods

### Development of the QOL-NPC

**Fig. 1:**
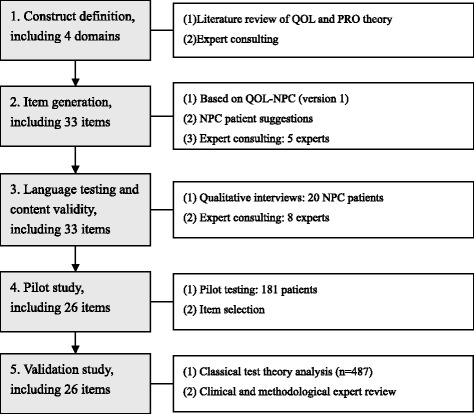
Steps towards development and validation procedure

The standard development and validation procedures were followed to develop and validate the QOL-NPC [19–23]. The procedures are shown in Fig. 1, which included construct definition, item generation, language testing and content validity, pilot study, and validation study.

### Construct definition and item generation

The QOL-NPC (V1) contains 30 items in four domains: physical function (PH, seven items); psychological function (PS, six items); social function (SO, five items); and side effect (SE, 12 items).

The domains of the QOL-NPC (V2) were sourced from V1. The items of the V1 were carefully discussed and revised by five experts. For example, the item “worried about the infection of the disease” was revised to “worried about the inheritance of the disease.” According to suggestions from NPC patients and clinical professionals, the following three items were added: have a pain in your throat (PH domain); cough when swallowing food (PH domain); and feel difficult to communicate with your family and friends (SO domain). Finally, a total of 33 items were generated. The VAS scale of the item was revised into a 1–5 Likert scale. The 1–5 Likert scales were expressed as not at all (excellent), a little bit (very good), moderate (good), quite a bit (fair), and extreme (poor).

### Language testing and content validity

All of the items were tested in a convenience sample of 20 NPC patients from different educational levels. The patients were asked whether or not they could understand the meaning of the items. Problematic items were revised according to the comments of the patients.

Eight experts were asked to assess the content validity. Expert consulting was available to evaluate whether or not the items of the QOL-NPC could represent the most relevant and important aspects of NPC patients [24]. Minor revisions and rewording of some items were performed until content validity was achieved.

### Pilot testing

A cross-sectional study (pilot testing) was conducted to select the items. A total of 181 NPC patients were enrolled. The Research Ethics Committee of the Cancer Center at Sun Yat-Sen University provided ethical approval. The sample size was 5–10 times the item number for the pilot test. The items were screened and selected using the floor and ceiling method, coefficient of variation, correlation analysis, internal consistency coefficients, and confirmatory factor analysis. According to item selection, seven items were deleted, five of which were deleted from the SE domain. Due to the popularity of intensity-modulated radiotherapy (IMRT), the NPC patients had fewer side effects, such as dysphonia, alopecia (hair loss), dizziness, and decreased vision due to RT.

After the item selection, the QOL-NPC (V2) contained 26 items in four domains: PH (eight items); PS (five items); SO (five items); and SE domain (eight items). Each item scored 1 to 5 points. Each domain was transformed into a 0–100 score. A higher score indicated a better QOL. The scale was self-administered by the patients. The scale was showed in Appendix 1.

### Validation study

A cross-sectional study (validation study) was conducted to assess the psychological characteristics of the QOL-NPC V2. The Research Ethics Committee of the Cancer Center at Sun Yat-Sen University provided ethical approval. The study was conducted between 1 July 2013 and 31 May 2014. Eligibility criteria included the following: (1) pathologically-proven NPC in the Cancer Center of Sun Yat-Sen University; (2) ≥16 years of age; and (3) able to provide informed consent to participate. The patients were excluded if diagnosed with another cancer, NPC relapse, or unconscious, confused, or cognitively impaired. The cognitively impaired were diagnosed by a psychologist.

The investigators included two medical post-graduates and three physicians, who were trained before the survey. The investigators explained the aim of our study before obtaining informed consent from the patients. If the patients agreed to participate in the survey, a questionnaire was given to them. The questionnaire included a socio-demographic sheet, QOL-NPC V2, FACT-G, and FACT-H&N. The socio-demographic sheet covered gender, educational degree, marriage status, dialect (Cantonese, Hakka, Chaoshanese, and others), pathologic type, Union for International Cancer Control (UICC) stage, methods of RT, RT stage, and other disease. The patients completed the questionnaire without assistance. If the patients did not understand the items on the questionnaire, the investigators explained them. If the questionnaire had missing data, the questionnaire was immediately returned to the patient for completion.

Terwee et al. considered a sample size of at least 50 patients to be adequate for the assessment of retest reliability and responsiveness [25]. Eighty inpatients were required to complete the QOL-NPC V2 within 2–3 days, which was used for the retest-test. A short interval (2–3 days) was chosen for the following reasons: (1) The NPC in-patients were all treated with RT, which had an obvious influence on QOL, especially for the long interval. (2) Marx et al. reported no significant differences for the test-retest reliability of 2 day and 2 week intervals [26]. The newly-diagnosed patients (60 patients), who had not been previously treated by RT, were required to finish the QOL-NPC after 50 ± 2 days of RT treatment. The data were used for the responsiveness test. These patients completed the scale by themselves in the retest and responsiveness tests. The investigators, the setting environment, and the investigation procedure were the same as the first test.

### Data analysis

Classical test theory (CTT) was used to assess the scale. SPSS 21.0 (Chicago, IL, USA) and Lisrel software (version 8.7) were performed [27]. The percentage of missing data, and the time to complete the instrument was calculated. Internal consistency reliability and split-half reliability were assessed using Cronbach's alpha value and Pearson's correlation coefficients between two halves of the items, respectively. Test-retest reliability was evaluated using an intra-class correlation coefficient (ICC) and the 95 % confidence interval (CI) of the two scores within 2–3 days. The correlation coefficients of the item-own domain (the item and its own domain) and the item-other domains (the item and other domains) were calculated. Construct validity was evaluated by the normed fit index (NFI), non-normed fit index (NNFI), comparative fit index (CFI), and root mean square error of approximation (RMSEA) based on confirmatory factor analysis (CFA) [28–30]. The correlation coefficients between the QOL-NPC and the FACT (FACT-G and FACT-H&N) were calculated to assess criterion validity. Discriminant validity was assessed by comparing the domain scores of the patients among different RT stages and different RT methods (analysis of variance). A paired samples *t*-test was used to analyze the score changes over time. Effect size was calculated as the change in scores divided by the standard deviation of the baseline score [31].

## Results

A sample of 500 patients was enrolled in the study. Thirteen patients (2.6 %) did not complete the questionnaire. Thus, 487 patients were included for the analysis (Table 1). The mean age was 47.0 ± 11.1 years (range, 16.1–78.1 years). There were 341 male and 146 female patients. Of the patients, 93.0 % were married, 68.8 % were Cantonese, 88.9 % were the undifferentiated type, 46.6 % were III stage, and 79.9 % did not have another disease.

**Table 1:** Characteristics of the patients

	Number (%, *n* = 487)	Number (%, *n* = 80)^a^	Number (%, *n* = 60)^b^
Gender			
Male	341 (70.0)	58 (72.5)	43 (71.7)
Female	146 (30.0)	22 (27.5)	17 (28.3)
Educational degree			
≤9 years	272 (55.9)	60 (75.0)	45 (75.0)
~12 years	180 (37.0)	14 (17.5)	12 (20.0)
>12 years	35 (7.2)	6 (7.5)	3 (5.0)
Marriage stage			
Unmarried	34 (7.0)	12 (15.0)	9 (15.0)
Married	453 (93.0)	68 (85.0)	51 (85.0)
Dialect			
Cantonese	335 (68.8)	53 (66.3)	38 (63.3)
Hakka	87 (17.9)	14 (17.5)	12 (20.0)
Chaoshanese	48 (9.9)	5 (6.3)	5 (8.3)
Others	17 (3.5)	8 (10.0)	5 (8.3)
Source of the patients			
In-patients	80 (16.4)	80 (100.0)	60 (100.0)
Out-patients	407 (83.6)	0 (0.0)	0 (0.0)
Pathological type			
Squamous cell	22 (4.5)	6 (7.5)	5 (8.3)
Differentiation type	32 (6.6)	9 (11.3)	5 (8.3)
Undifferentiated type	433 (88.9)	65 (81.3)	50 (83.3)
UICC stage			
I stage	22 (4.5)	0 (0.0)	0 (0.0)
II stage	78 (16.0)	12 (15.0)	4 (6.7)
III stage	227 (46.6)	36 (45.0)	28 (46.7)
IV stage	160 (32.9)	32 (40.0)	28 (46.7)
Radiotherapy			
IMRT	294 (60.4)	72 (90.0)	60 (100.0)
Three-dimensional RT	47 (9.7)	2 (2.5)	0 (0.0)
Conventional RT	86 (17.7)	4 (5.0)	0 (0.0)
No	60 (12.3)	2 (2.5)	0 (0.0)
Other disease			
Yes	98 (20.1)	60 (75.0)	47 (78.3)
No	389 (79.9)	20 (25.0)	13 (21.7)

^a^The patients were used for the retest test

^b^The patients were used for the responsiveness test

The average time to complete the instrument was 8.4 ± 4.6 min, ranging from 3.8 to 16.3 min. Ten patients did not understand certain items, such as the item “mental stress”. They completed the items with the help of the investigators. The scores of all the items ranged from 1 to 5 (Table 2). Item SE8 scored the highest (3.90), while item PS2 scored the lowest (2.46). Item SE8 had 3.7 % missing data. Other items had a lower proportion of missing data.

**Table 2:** Missing data, mean and SD for each item (*n* = 487)

	Score 1	Score 2	Score 3	Score 4	Score 5	Missing (%)	Mean	SD
PH1	13	92	195	170	13	4(0.8)	3.16	0.86
PH2	24	105	170	161	20	7(1.4)	3.10	0.96
PH3	28	184	191	60	10	14(2.9)	2.66	0.85
PH4	14	63	155	166	82	7(1.4)	3.50	1.02
PH5	13	40	96	209	125	4(0.8)	3.81	1.00
PH6	20	60	101	171	129	6(1.2)	3.68	1.12
PH7	14	56	83	213	117	4(0.8)	3.75	1.04
PH8	16	70	129	183	86	3(0.6)	3.52	1.05
PS1	5	30	146	236	63	7(1.4)	3.67	0.82
PS2	33	281	101	59	12	1(0.2)	2.46	0.88
PS3	48	235	64	113	23	4(0.8)	2.64	1.09
PS4	35	207	168	59	16	2(0.4)	2.62	0.91
PS5	10	26	116	293	39	3(0.6)	3.67	0.78
SO1	8	17	122	281	55	4(0.8)	3.74	0.77
SO2	5	40	86	261	92	3(0.6)	3.82	0.87
SO3	38	158	63	160	67	1(0.2)	3.12	1.23
SO4	34	217	95	107	31	3(0.6)	2.76	1.07
SO5	5	46	184	220	28	4(0.8)	3.46	0.79
SE1	32	117	182	136	17	3(0.6)	2.98	0.96
SE2	31	76	141	147	87	5(1.0)	3.38	1.14
SE3	11	41	122	205	98	10(2.1)	3.71	0.96
SE4	7	38	111	184	138	9(1.8)	3.85	0.98
SE5	11	62	105	192	105	12(2.5)	3.67	1.03
SE6	14	62	126	181	93	11(2.3)	3.58	1.04
SE7	9	55	138	168	102	15(3.1)	3.63	1.01
SE8	6	34	96	197	136	18(3.7)	3.90	0.94

*PHx* the xth item of the PH domain, *score 1* lowest QOL, *score 5* highest QOL, *SD* standard deviation

The mean score of the SE domain was the maximum (64.5), and the mean score of the PS domain was the minimum (50.3; Table 3). The Cronbach's alpha value of the domain ranged from 0.72 to 0.84. The split-half coefficients of the domain ranged from 0.77 to 0.84. The SE domain had a maximum Cronbach's alpha value and split-half coefficient. All of the ICCs were >0.8, and of all the coefficients were significantly different.

**Table 3:** Descriptive statistics and reliability of the QOL-NPC (*n* = 487)

Domain	No. of items	Mean ± SD	Range of score	Cronbach’s alpha	Split-half coefficient	ICC (95 % CI)
PH	8	60.0 ± 16.9	(6.3, 100.0)	0.80	0.83	0.87 (0.85, 0.89)
PS	5	50.3 ± 15.5	(0.0, 100.0)	0.72	0.77	0.88 (0.86, 0.90)
SO	5	59.4 ± 17.2	(0.0, 100.0)	0.76	0.81	0.82 (0.78, 0.86)
SE	8	64.5 ± 17.6	(3.1, 100.0)	0.84	0.84	0.88 (0.84, 0.92)

All items correlated more strongly with their own domain than the other domains (Table 4). For example, the correlation coefficients of the items in the PH domain and PH ranged from 0.47 to 0.77, which were greater than the other domains.

**Table 4:** Items-domains correlation analysis of QOL-NPC (*n* = 487)

	Correlation with item				Factor loading of CFA
	PH	PS	SO	SE	
PH1	0.54	0.31	0.27	0.29	0.67
PH2	0.72	0.33	0.34	0.39	0.72
PH3	0.64	0.30	0.34	0.39	0.66
PH4	0.63	0.15	0.29	0.45	0.59
PH5	0.48	0.09	0.23	0.29	0.51
PH6	0.77	0.29	0.22	0.55	0.65
PH7	0.47	0.22	0.19	0.32	0.51
PH8	0.64	0.20	0.21	0.44	0.55
PS1	0.36	0.61	0.42	0.35	0.53
PS2	0.22	0.79	0.35	0.26	0.72
PS3	0.17	0.74	0.25	0.20	0.57
PS4	0.37	0.80	0.40	0.37	0.81
PS5	0.22	0.53	0.27	0.16	0.54
SO1	0.21	0.26	0.58	0.17	0.47
SO2	0.26	0.24	0.61	0.31	0.47
SO3	0.19	0.31	0.80	0.21	0.63
SO4	0.38	0.45	0.78	0.28	0.74
SO5	0.41	0.43	0.71	0.33	0.70
SE1	0.52	0.32	0.22	0.65	0.63
SE2	0.71	0.33	0.24	0.74	0.76
SE3	0.46	0.26	0.24	0.71	0.66
SE4	0.45	0.25	0.28	0.70	0.65
SE5	0.31	0.16	0.21	0.69	0.56
SE6	0.25	0.20	0.26	0.63	0.50
SE7	0.47	0.35	0.34	0.73	0.66
SE8	0.41	0.29	0.27	0.68	0.59

**Fig. 2:**
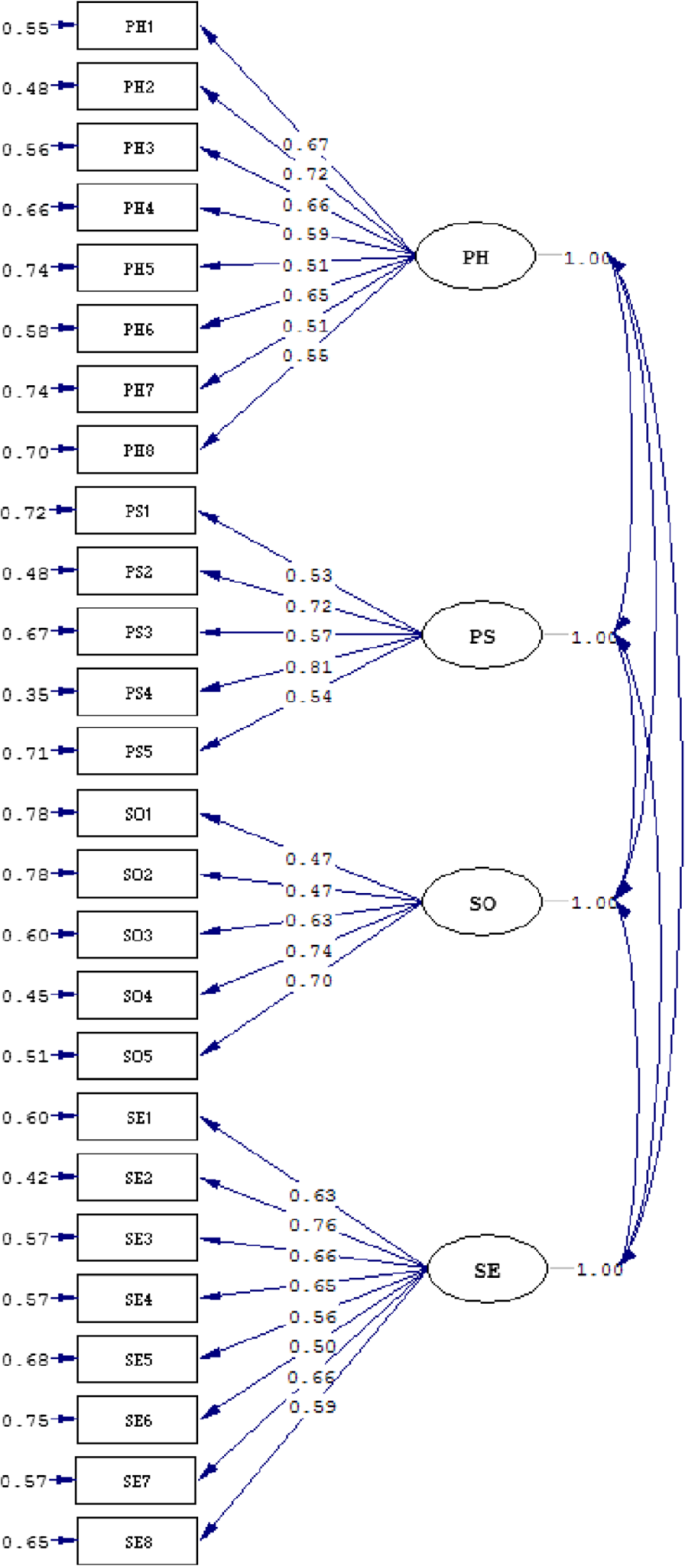
Results of CFA

The results of the CFA analysis showed that the RMSEA was equal to 0.097 with a 90 % CI (0.093, 0.100). Both the NFI and NNFI were equal to 0.89. The CFI was equal to 0.90. The factor loadings of CFA are shown in Table 4. The minimum factor loading was 0.47 (SO1 and SO2). The structure diagram is shown in Fig. 2.

The PH, PS, and SO domains of the QOL-NPC had a positive correlation with physical, emotional, and social/family well-being of the FACT-G with coefficients of 0.71, 0.63, and 0.56, respectively. The correlation coefficient between the SE domain of the QOL-NPC and FACT-H&N was 0.51, which was significantly different.

The PH, PS, and SE domain scores of patients in different RT stages were significantly different (*P* <0.001; Table 5). The patients who were receiving RT had the lowest scores in the PH, PS, and SE domains. The patients before RT and >5 years after RT had the highest scores. The SO domain scores of patients in different RT stages were not significantly different (*P* >0.05). All the domain scores of patients using different RT methods were significantly different (*P* <0.001; Table 5). The patients who did not receive RT had the highest scores, followed by those receiving intensity-modulated radiotherapy (IMRT).

**Table 5:** The domain scores (mean ± SD) of patients among different RT stages and RT method

	n	PH	PS	SO	SE
RT stages					
Before RT	60	65.9 ± 15.5	54.5 ± 15.8	60.8 ± 17.6	71.6 ± 17.0
During RT	171	54.0 ± 14.9	47.4 ± 13.6	58.3 ± 15.7	59.2 ± 17.9
≤1 year after RT	145	59.3 ± 14.2	48.5 ± 16.5	57.5 ± 15.6	62.6 ± 16.3
~5 years after RT	65	63.5 ± 14.1	55.1 ± 16.3	63.8 ± 17.6	70.5 ± 15.9
>5 years after RT	46	72.3 ± 17.4	54.3 ± 14.7	61.3 ± 24.2	73.0 ± 15.4
*P*-value		<0.001	<0.001	0.100	<0.001
RT method					
IMRT	294	61.3 ± 15.7	51.5 ± 15.2	61.8 ± 16.4	66.8 ± 16.7
Three-dimensional RT	46	56.8 ± 13.2	44.5 ± 14.1	56.7 ± 17.1	60.6 ± 17.5
Conventional RT	86	53.4 ± 16.1	46.3 ± 15.6	51.8 ± 17.7	53.9 ± 16.6
No RT	60	65.9 ± 15.5	54.5 ± 15.8	60.8 ± 17.6	71.6 ± 17.0
*P*-value		<0.001	<0.001	<0.001	<0.001

*RT* radiotherapy, *IMRT* intensity-modulated radiotherapy

Sixty patients before RT were enrolled to test the responsiveness of the QOL-NPC over time. At the completion of RT, all domain scores decreased (Table 6). The score changes for all domains were significantly different, with effect sizes ranging from −0.82 (SE domain) to −0.22 (SO domain).

**Table 6:** Change in scores and effect size from baseline to the end of radiotherapy (*n* = 60)

	Baseline score	Change scores^a^	95 % CI of change scores	Effect size^b^
PH	65.9 ± 15.5	−4.9 ± 6.4	(3.2, 6.5)	−0.31
PS	54.5 ± 15.8	−3.8 ± 5.3	(2.4, 5.2)	−0.24
SO	60.8 ± 17.6	−3.9 ± 6.0	(2.4, 5.5)	−0.22
SE	71.6 ± 17.0	−5.9 ± 5.4	(4.5, 7.3)	−0.82

Change scores^a^, the score at the end of radiotherapy minus the baseline, −100 (maximum worsening) to +100 (maximum improvement)

Effect size^b^, calculated as the change in scores divided by the SD of the baseline score

## Discussion

The most commonly specific instruments used to assess QOL of NPC patients include QLQ-C30, QLQ-H&N35 [4, 32, 33], and FACT-NP, which consists of FACT-G and NPC subscale [11]; however, due to cultural differences, we developed the QOL-NPC to assess the QOL of Chinese NPC patients.

The QOL-NPC (V2) had good content validity according to the suggestions of experts. The QOL-NPC was broadly defined as the endpoint directly derived from the patient, which included symptoms, health status, adherence, and side effect [34]. The QOL-NPC included the most important aspects characterizing specific aspects of NPC patients, which is structurally made up of physical function, psychological function, social function, and side effects. For example, physical function included feeling tired, losing weight, having a headache, nasal tampon or nasal bleeding, satisfied with appearance, and coughing when swallowing food. Side effects included dry mouth (xerostomia), pain in the throat, difficulty in opening the mouth, memory decline, skin injuries in the head and neck, and damaged teeth due to RT. It is well-accepted that dry mouth is the most significant morbidity during and following RT, which causes serious disorders in tasting, chewing, and swallowing, as well as sleeping disorders [35].

The QOL-NPC (V2) had good reliability and validity based on the results of CTT. All of the domains had moderate or high Cronbach's alpha coefficients (0.72–0.84), and split-half reliability coefficients (0.77–0.84). The researchers gave a positive rating for internal consistency when Cronbach's alpha was >0.70 [25]. All of the domains had high intra-class correlation coefficients (0.82–0.88), which indicated that the QOL-NPC (V2) can evaluate the QOL of patients. Based on the results of CFA, RMSEA was equal to 0.097 with a 90 % CI (0.093, 0.100); NFI, NNFI, and CFI approached 0.90. The factor loadings of CFA were >0.47. These results showed that the QOL-NPC had good construct validity. The corresponding domains of the QOL-NPC and the FACT-G and FACT-H&N were significantly related to each other. For example, the PH domain of the QOL-NPC had a significantly positive correlation with physical well-being of the FACT-G.

The PH, PS, and SE domain of the QOL-NPC were sensitive to discriminate the QOL of NPC patients in different RT stages. The QOL of the NPC patients during RT were the lowest. These results were consistent with our hypothesis. It is known that RT has a serious impact on the health status of the patients [4, 36]. All the domain scores of patients using different RT methods were significantly different. The patients who did not receive RT had the highest scores. The patients who receive IMRT had the higher scores than those receiving other RT methods. Our results were consistent with other studies, which showed that IMRT played a significant role in improving the QOL of NPC patients [37, 38].

The QOL-NPC (V2) had good responsiveness based on the results of CTT. After the newly diagnosed NPC patients received RT treatment, they had numerous side effects, especially head and neck symptoms, such as pain in the mouth and throat, dry mouth, and difficulties in speaking. Therefore, the domain scores of the QOL-NPC decreased. The effect sizes of these domains ranged from −0.82 to −0.22. The effect sizes of the SE domain were greater than the other domains. Similar decreases in QOL were observed in the Quality Of Life Radiation Therapy Instrument and the Head & Neck Module [39].

The QOL-NPC had good operability. Of the patients, 97.4 % completed the questionnaire. Most of the items had a lower proportion of missing data. The patients completed the QOL-NPC in an average time of 8.4 min.

There were some study limitations. (1) All of the patients in the study were enrolled from the Cancer Center of Sun Yat-Sen University. The QOL-NPC (V2) should be further evaluated by the data from other centers. (2) Only 60 NPC patients were used to test the responsiveness of the QOL-NPC (V2). The responsiveness of the scale should be further assessed in a larger sample of patients. (4) Some patients completed the QOL-NPC with the help of the investigators. It was a limitation of the study, for item explanation by a third party can generate application bias.

## Conclusions

The QOL-NPC (V2) is valid for measuring QOL with good reliability, validity, and responsiveness. We recommend the application of the QOL-NPC (V2) for measuring QOL in the Chinese NPC patients.

## Appendix 1

Quality of life scale of nasopharyngeal carcinoma patients: The QOL-NPC (version 2).

The Quality of life scale of nasopharyngeal carcinoma patients (version 2) includes 26 items, which is presented as a five-point scale. Please respond to each item according to your feeling in the past 2 weeks. The following items are about your physical function, psychological function and social function related to nasopharyngeal carcinoma.

**Table:**

PH1. Sleep	poor	fair	good	very good	excellent
PH2. Appetite	poor	fair	good	very good	excellent
PH3. Satisfaction with appearance	not at all	a little bit	moderate	quite a bit	extreme
PH4. Weight loss	extreme	quite a bit	moderate	a little bit	not at all
PH5. Tiredness	extreme	quite a bit	moderate	a little bit	not at all
PH6. Headache	extreme	quite a bit	moderate	a little bit	not at all
PH7. Nasal tampon or nasal bleeding	extreme	quite a bit	moderate	a little bit	not at all
PH8. Coughing while swallowing	extreme	quite a bit	moderate	a little bit	not at all
PS1. Feeling lives to be meaningful	not at all	a little bit	moderate	quite a bit	extreme
PS2. Satisfaction with treatment effect	not at all	a little bit	moderate	quite a bit	extreme
PS3. Worries about impact of disease	extreme	quite a bit	moderate	a little bit	not at all
PS4. Worries about inheritance of disease	extreme	quite a bit	moderate	a little bit	not at all
PS5. Mental stress	extreme	quite a bit	moderate	a little bit	not at all
SO1. Satisfaction with interpersonal relations	not at all	a little bit	moderate	quite a bit	extreme
SO2. Satisfaction with ability of work and daily life	not at all	a little bit	moderate	quite a bit	extreme
SO3. Difficulty in communication	extreme	quite a bit	moderate	a little bit	not at all
SO4. Influence of role in family and work	extreme	quite a bit	moderate	a little bit	not at all
SO5. Influence of social activities	extreme	quite a bit	moderate	a little bit	not at all

The following items were about the side effects due to radiotherapy.

**Table:**

SE1. Dry mouth	extreme	quite a bit	moderate	a little bit	not at all
SE2. Throat pain	extreme	quite a bit	moderate	a little bit	not at all
SE3. Harmfulness in head-neck skin	extreme	quite a bit	moderate	a little bit	not at all
SE4. Difficulty in opening mouth	extreme	quite a bit	moderate	a little bit	not at all
SE5. Loosed and damaged teeth	extreme	quite a bit	moderate	a little bit	not at all
SE6. Hearing loss	extreme	quite a bit	moderate	a little bit	not at all
SE7. Radioactive rhinitis	extreme	quite a bit	moderate	a little bit	not at all
SE8. Memory loss	extreme	quite a bit	moderate	a little bit	not at all

## Abbreviations

CI: confidence interval
CFA: confirmatory factor analysis
CFI: comparative fit index
CTT: classical test theory
ICC: intra-class correlation coefficient
NFI: normed fit index
NNFI: non-normed fit index
RMSEA: root mean square error of approximation
FACT-G: Functional Assessment of Cancer Therapy-General Scale
FACT- H&N: Functional Assessment of Cancer Therapy head and neck module
FACT-NP: the functional assessment of cancer therapy-nasopharyngeal
IMRT: intensity-modulated radiotherapy
QOL-NPC: quality of life scale of nasopharyngeal carcinoma patients
RT: radiotherapy
NPC: nasopharyngeal carcinoma
QOL: quality of life
QLQ-C30: the research and treatment of cancer core QOL questionnaire
QLQ-H&N35: the research and treatment of cancer questionnaire head and neck module
PH: physical function
PS: psychological function
SO: social function
SE: side effect
UICC: Union for International Cancer Control
VAS: visual analogue scale
V1: version 1
V2: version 2
